# Metal–Organic Frameworks-Based Microrockets for Controlled and Sustained Drug Release

**DOI:** 10.1021/acs.nanolett.4c04628

**Published:** 2025-03-17

**Authors:** Zixi Wan, Casper H.Y. Chung, Chi Ming Laurence Lau, Jin Teng Chung, Ying Chau, Zhiyong Fan, Shuaizhong Zhang, Shuhuai Yao

**Affiliations:** † Department of Mechanical and Aerospace Engineering, The Hong Kong University of Science and Technology, Hong Kong, SAR, China; ‡ Department of Chemical and Biological Engineering, The Hong Kong University of Science and Technology, Hong Kong, SAR, China; § Department of Electronic and Computer Engineering, The Hong Kong University of Science and Technology, Hong Kong, SAR, China

**Keywords:** metal−organic frameworks (MOFs), microrockets, pH-response, drug delivery, controlled and sustained release

## Abstract

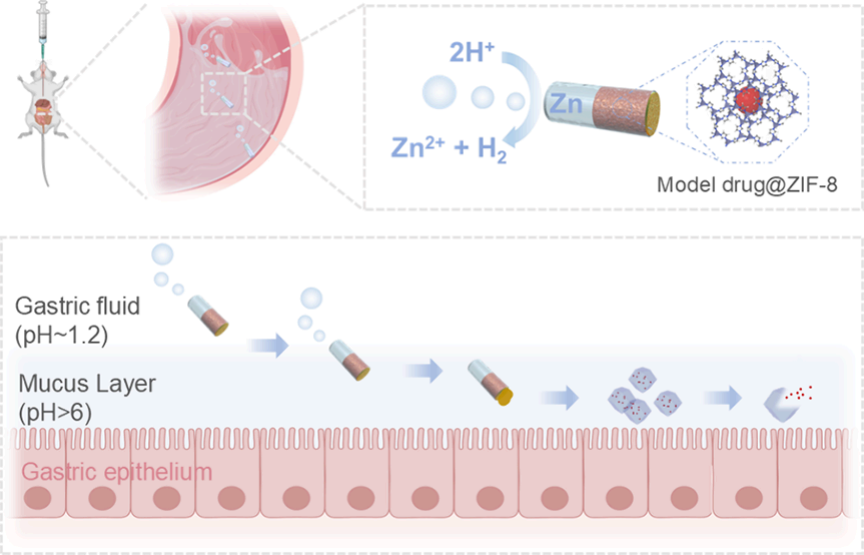

Gastritis, linked to chronic stress and poor diets, poses significant risks, including ulcers and gastric cancers. Current treatments involving frequent dosing of multiple drugs face challenges with patient nonadherence and antibiotic resistance. To overcome these issues, metal–organic framework (MOF)-based microrockets utilizing a zinc-powered engine were engineered with pH-sensitive coatings for targeted gastritis treatment. These microrockets can self-propel into the gastric mucus via bubble propulsion and slowly release pharmaceutical ingredients from MOF components. A poly(3,4-ethylenedioxythiophene) shell and pH-sensitive enteric coating is designed for protection of MOFs in acid while allowing sustained drug release at the mucosa’s neutral pH. In vivo studies demonstrate these microrockets sustain a prolonged drug retention for 48 h. This biocompatible design represents a promising strategy for active and controlled drug delivery with sustained release in acidic environments, presenting the potential for diverse biomedical applications.

Gastritis, inflammation of the stomach lining, is growing more common due to factors such as elevated stress levels and unhealthy dietary habits.^1−3^ This disease can lead to severe complications, including gastric ulcers and even gastric cancers if left untreated.^4,5^ Typical therapy has not been straightforward, mainly because of the harsh gastric acidic environment and the protective mucus layer that make it difficult for single antibiotics to effectively eradicate infections. Common treatments, such as triple therapy, require multiple medications taken frequently, typically 3 to 4 times per day over a course of 10 to 14 days.^6−10^ The success of these treatments relies on maintaining therapeutic drug levels, which means adhering strictly to the dosing schedule. Missing doses can lead to both treatment failure and antibiotic resistance.^9,10^ Consequently, there is a pressing need for innovative, sustained drug delivery systems that can reduce the dosing frequency and improve patient adherence, as well as treatment outcomes.

Metal–organic frameworks (MOFs), with their tunable crystalline and highly porous structure, offer a promising solution for sustained drug release.^11−13^ Their expansive surface area and high porosity facilitate efficient drug loading and sustained release.^14,15^ Additionally, most MOF materials (e.g., ZIF-8, MIL-100) are reported to be nontoxic and biocompatible under certain concentrations in preclinical level, enhancing their appeal for biomedical applications.^16,17^ MOFs have been utilized as drug carriers, and their sustained release capabilities have been explored to mitigate the side effects caused by traditional drugs, such as excess accumulation in healthy tissues.^18^ Drugs and other cargoes (e.g., protein, peptides, etc.) can be effectively loaded onto or within MOFs through surface adsorption, pore encapsulation, covalent binding, and the confinement effect.^18−21^ These cargo-loaded MOFs can release their contents in response to various active or passive triggers, such as thermo-stimuli, pH-stimuli, cargo desorption, and structure decomposition.^21−23^ To date, cargo-loaded MOFs have shown considerable promise in antibacterial and antitumor therapies.^18,24^ However, MOFs are vulnerable to degradation in the acidic gastric environment, limiting their use in oral drug delivery.^3,25^ To address this, self-propelled microrockets were involved here to transport drug-loaded MOFs directly to targeted areas within the mucosal layer (pH ≈ 7).^5,25,26^ Unlike externally propelled microrockets driven by light,^27^ electrical,^28^ acoustic,^29^ and magnetic fields,^30^ self-propelled microrockets convert chemical energy from chemical fuels,^31,32^ enzymatic reactions,^31,33^ or the Marangoni effect^34^ into kinetic energy. Among these autonomous microrockets, metal (e.g., zinc or magnesium) incorporated microrockets can gain sufficient mobility in aqueous biofluids, where abundant protons are present, especially in a gastric acid environment.^35−38^ Such microrockets move forward through detaching or bursting bubbles, which are generated through reactions between the metal fuels and the aqueous fluids.^39,40^ They are built into an asymmetrical structure to guide the bubbles to discharge directionally, which are commonly divided into Janus particles and tubular structures.^39^ For instance, Li et al. reported a magnesium-based Janus microsphere for autonomously releasing encapsulated cargoes in the stomach upon gastric acid neutralization.^41^ Rodolfo et al. developed zinc microrocket pills, which dissolve in the stomach and release the microrockets penetrating into the firm mucosal layer at a neutral pH characterized by a slower regeneration rate. Such actions have been demonstrated to enhance both the retention and controlled delivery of cargoes within the gastrointestinal (GI) tract.^35,42,43^

In this work, the joint merits of self-propulsion of microrockets and slow release of MOFs in multicompartment configuration are combined to achieve controlled and sustained drug delivery. The microrockets are made of a poly(3,4-ethylenedioxythiophene) (PEDOT) shell, featuring a zinc (Zn)-powered engine at one end and a pH-sensitive enteric coating protecting the drug-loaded MOFs at the other end. When the Zn fuel reacts with the gastric lumen’s hydrochloric acid, it enables directional propulsion of the microrockets to embed within the mucosal layer by producing hydrogen bubbles. The pH-sensitive enteric coating can survive strong acidic conditions to protect antibiotic-laden MOFs from being degraded by gastric acid while dissolving under the neutral pH condition of the stomach mucus layer. With zinc propulsion and pH-responsive coating, these microrockets can effectively deliver the drug-laden MOFs to a neutral mucosal area, thereby stimulating the drug’s effect without the need for proton pump inhibitors (PPIs). Through the MOFs’ gradual desorption and decomposition, drugs can be released into the targeted locations over a duration of several days. The prolonged release of the medical agents reduces the frequency of dosing required for effective gastritis therapy. Our proof-of-concept demonstration via in vitro and in vivo experiments shows a more uniform and steady distribution of delivered cargoes, proving that the MOF-encapsulated microrockets are promising for biomedical applications in harsh environments.

## Design and Working Principle of the MOF-Based Microrockets

**Figure 1:**
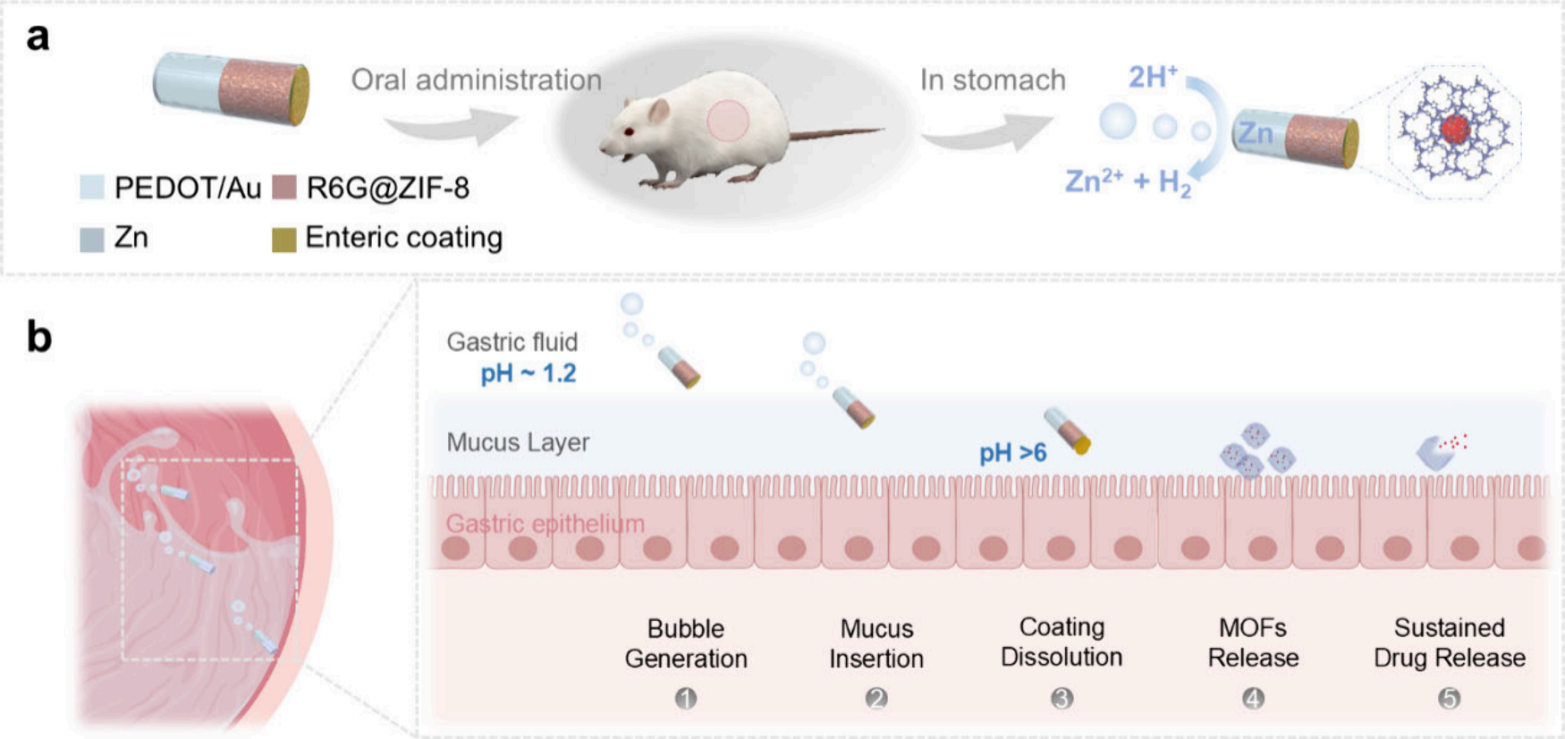
Schematic illustration of the self-propelled microrockets for controlled and sustained drug delivery. (a) The structure of the MOF-based microrockets: PEDOT/Au shell (lightblue), Zn engine (gray), model drug@ZIF-8 (red), and enteric coating (yellow); and their propelling mechanism: Zn + 2H^+^ = Zn^2+^ + H_2_. (b) Working mechanism of controlled and sustained drug release: (1) bubble generation from reaction with acid fluid, (2) mucus insertion resulting from the bubble-induced thrust, (3) enteric coating dissolution at a neutral pH condition, and (4) decomposition of zeolite imidazole frameworks (ZIF-8), a type of MOFs, leading to (5) sustained drug release in the mucosal layer of the stomach.

A sequential template-based electrodeposition method was developed to create microrockets. The microrockets are composed of a PEDOT/Au shell featured with a Zn-powered engine at one end and cargo-loaded MOFs within gelatin coated by a pH-sensitive enteric polymer at the other end (Figure 1a). The PEDOT shell can enhance the chemical and mechanical stability of the microrockets as well as protect the drug-loaded MOFs from harsh environments. The Zn compartment is designed as the engine to propel the microrockets by reacting with acids. Model drug@ZIF-8 is designed to achieve sustained drug release upon reaching the mucosa layer. The pH-sensitive enteric coating made of Eudragit L100 polymer, together with the PEDOT shell, protects the MOFs from being degraded in acidic conditions.

The working principle of our self-propelled microrockets centers on controlled and sustained drug delivery. Upon oral administration, exposure to acidic fluid activates the microrockets, triggering a spontaneous redox reaction at the Zn surface that results in the production of a hydrogen-bubble tail.^44^ The microbubble generation provides a robust propulsion thrust, propelling the microrockets swiftly through the gastric lumen. Such dynamic propulsion could enable a uniform distribution and an enhanced retention of the microrockets in the gastric mucus layer, thereby facilitating an improved and controlled delivery of cargoes in the deeper mucosa areas where the pH is about neutral. Upon reaching the region, the pH-sensitive coating dissolves, revealing ZIF-8 drug carriers and initiating the drug release process (Figure 1b). The drug components are sustainedly released from ZIF-8 structures due to the adsorption and degradation of MOFs in neutral biological environments, enabling a sustained therapeutic effect. Overall, our MOF-based microrockets offer a promising solution for prolonged drug retention under harsh conditions due to controlled and sustained drug release. This is achieved through the synergy of self-propelled microrockets and MOF carriers, demonstrating the potential for innovative treatments of gastric diseases such as gastritis.

## Fabrication and Characterization of the MOF-Based Microrockets

**Figure 2:**
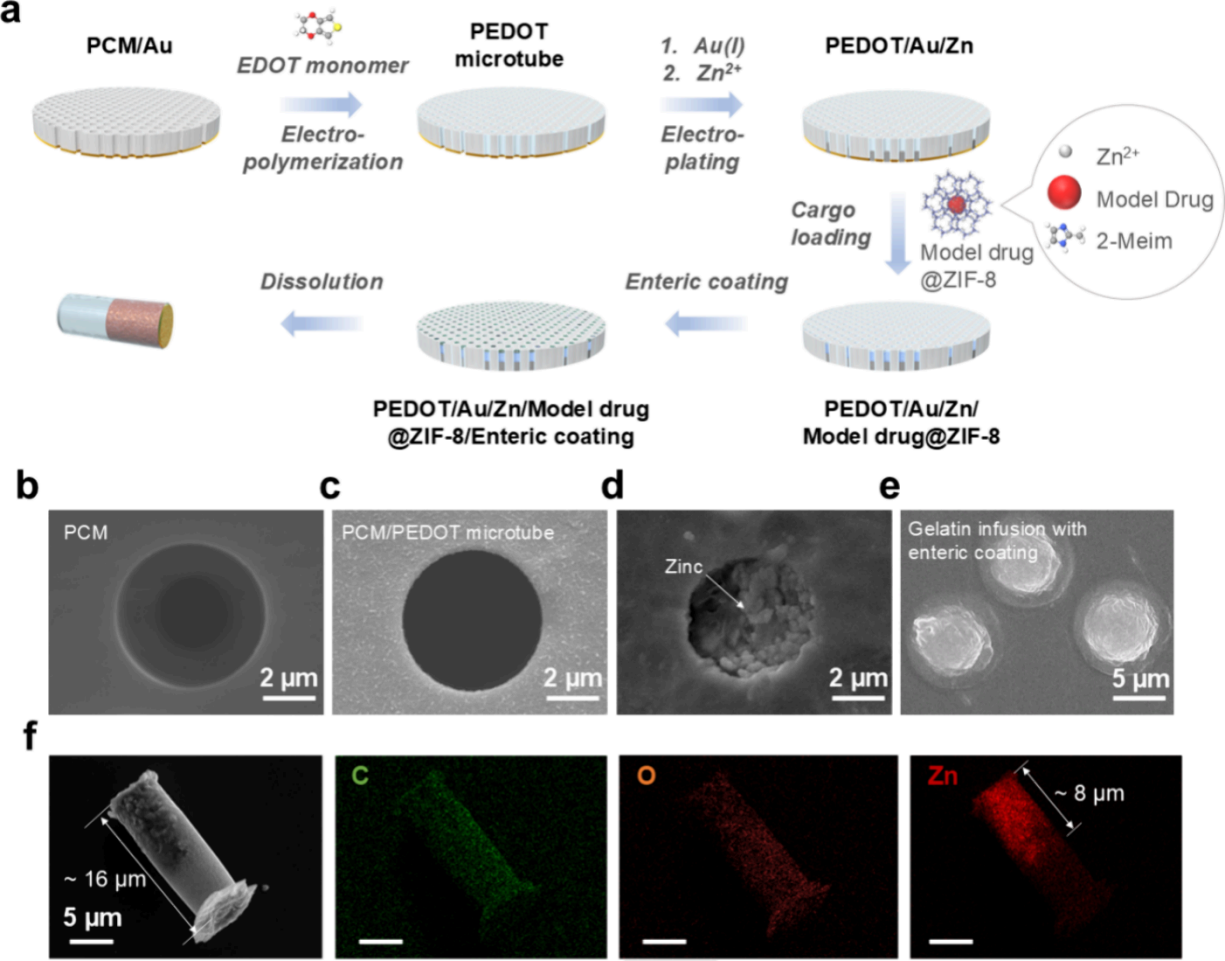
Fabrication and characterization of MOF-based microrockets. (a) Schematic illustration of the template-based fabrication process for the microrockets loaded with model drug@ZIF-8: (1) PEDOT is electro-polymerized into the micropores of a PCM template coated with Au on the bottom side, (2) Au shell and Zn end are electrodeposited onto the structure, (3) the model drug@ZIF-8 is loaded into the microrockets, (4) An enteric coating is applied, and (5) the PCM template is dissolved to release the microrockets. SEM images of the microrockets at different fabrication stages: (b) the bare PCM template, (c) the PCM template with PEDOT polymerized onto the channel walls, (d) Zn deposited from the bottom side of the template, and (e) model drug-loaded ZIF-8 within gelatin infusion and enteric coating on top of the template. (f) A complete microrocket along with EDX images illustrating the distribution of elemental carbon (green), oxygen (orange), and zinc (red).

The microrockets were prepared using a template-assisted fabrication method (Figure 2a).^45,46^ Polycarbonate membranes (PCM) with 5 μm columnar pores were used as the template. First, a 75 nm layer of gold (Au) was evaporated on the template, serving as the working electrode for the electrodeposition of the PEDOT/Au microshell. Subsequently, PEDOT shells of approximately 200 nm and Au tubes were electrodeposited within the micropores. The thin gold layer not only enhances the robustness of the PEDOT/Au microshells but facilitates the integration of the Zn compartment and cargo loading. Following the gold plating, the Zn compartment was electrodeposited from the bottom of the template. The length of the Zn engine, which can be controlled by adjusting the deposition duration and input current, allows for the flexible tuning of the microrockets’ lifetime and drug loading capacity. After Zn deposition, the bottom of the template was polished by using ion beam milling to remove the residual gold and excess Zn. A mixture of model drug-loaded ZIF-8 and gelatin was then transfused into the template, followed by an enteric coating step.^47^ Rhodamine 6G (R6G) and IRDye 800CW carboxylate were selected as model drugs because they allow for the visualization of drug distribution with small particle sizes comparable to those of commonly used medications for treating gastritis, such as amoxicillin. The model drug-loaded ZIF-8 particles were synthesized through a one-pot synthesis method and the cargoes were encapsulated in and adsorbed on ZIF-8 particles.^48^ Finally, the self-propelled microrockets were released by dissolving the PCM template with dichloromethane (DCM) and rinsing it with ethanol. Further details of the fabrication process are available in the Supporting Information.

Scanning electron microscopy (SEM) images were taken at each fabrication stage to validate our fabrication method (Figures 2b–2e). These SEM images clearly confirm the successful fabrication of each component of the microrockets within the PCM template: PEDOT microtubes, Zinc end, and cargo-loaded MOFs within gelatin coated with enteric cap. Thin yet robust PEDOT microshells were formed (Figure 2c) within the uniform and columnar pores of the PCM (Figure 2b). The bottom view of PCM reveals the rough and dense zinc end after the electrodeposition (Figure 2d), whereas the top view shows a smooth surface after infusion of cargo-loaded MOFs within the gelatin and the enteric coating (Figure 2e). The SEM image and energy-dispersive X-ray spectroscopy (EDX) display a segmented deposition of zinc, approximately 8 μm in length, adjustable by varying the electrodeposition duration (Figure 2f and Figure S1). The microrockets, consistent in size at approximately 16 μm in length and 5 μm in diameter, demonstrate reproducible fabrication (Figure S2a) and feature a uniform, well zoned structure with a protective enteric coating (Figure S2b). This coating, made of Eudragit L100 polymer, is a pH-responsive material that remains stable in acidic environments while dissolving under neutral pH conditions. With this protective layer, the MOFs filled in the microrockets can survive the harsh acids while smoothly releasing the cargoes where the pH is about neutral.

## Zinc-Powered Propulsion of Microrockets

**Figure 3:**
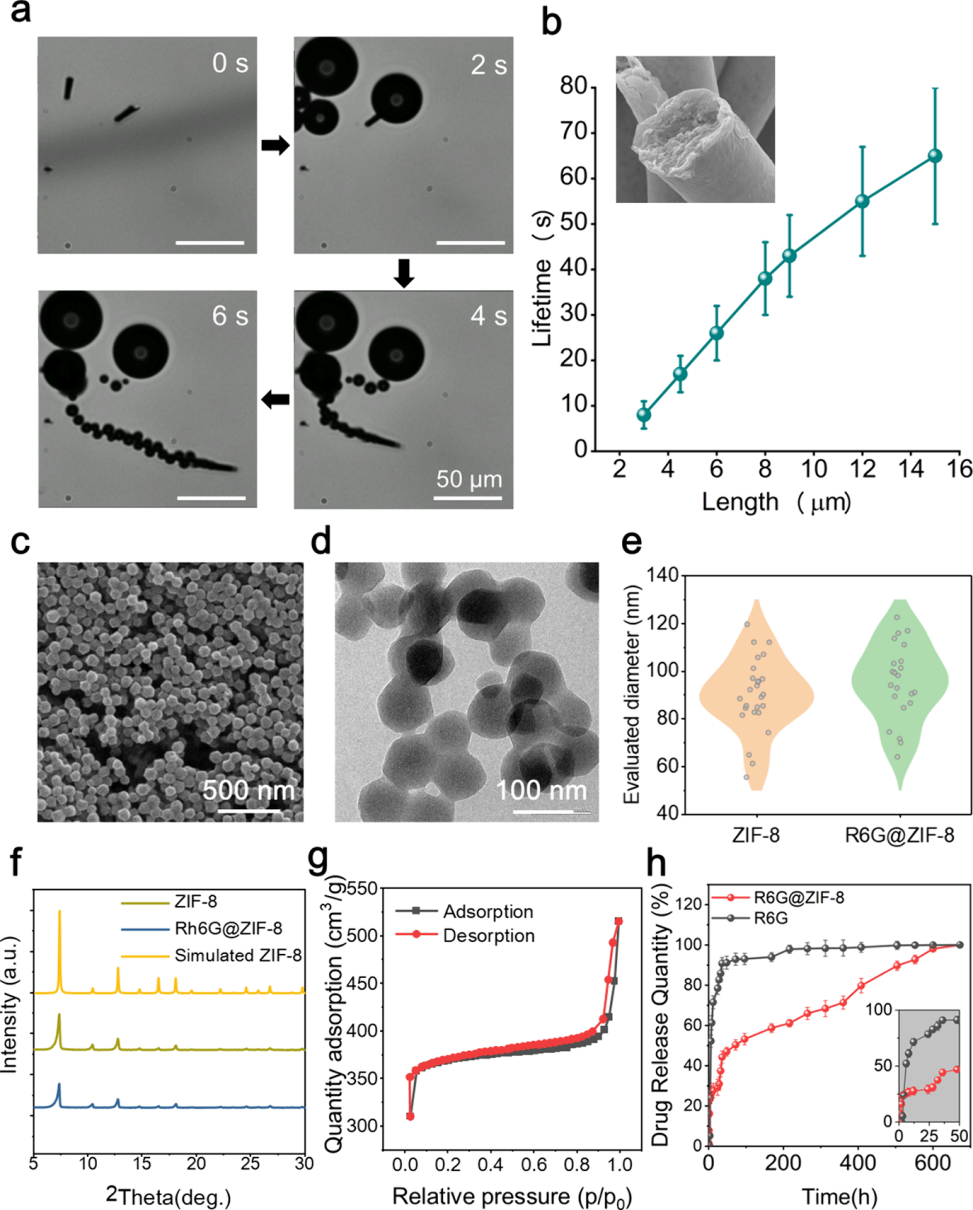
Self-propulsion and sustained drug delivery capabilities of the as-developed microrockets. (a) Time-lapse images of the microrockets showing their self-propulsion in a simulated gastric fluid (pH = 1.2). (b) Lifetime of the microrockets as a function of the Zn segment length in the simulated gastric fluid. Inset is an SEM image showing a cross-sectional view of the Zn segment. (c) SEM images of R6G@ZIF-8 nanoparticles. (d) TEM image of R6G@ZIF-8 nanoparticles. (e) Size distribution of ZIF-8 and R6G@ZIF-8 nanoparticles extracted and calculated from the SEM images. (f) XRD patterns of ZIF-8, R6G@ZIF-8, and simulated ZIF-8 crystals. (g) N_2_ adsorption–desorption isotherm curves of ZIF-8 nanoparticles. (h) Comparison of model drug release with and without ZIF-8 in a PBS solution.

To evaluate the propulsion of our Zn-powered microrockets, we tested them in simulated gastric fluid at a pH of 1.2. Time-lapse imaging captured every two seconds, as shown in Figure 3a and Video S1, demonstrated bubble generation and rapid self-propulsion at speeds of 40 μm/s. This propulsion is crucial for transversing the mucus barrier to ensure an efficient drug retention. The lifetime of the microrockets depends on the length of the Zn-loaded compartment. The impact of variations in the Zn-loaded compartment length on lifespan and drug loading capacity was thoroughly investigated. As illustrated in Figure 3b, the average lifespan of the microrockets in gastric fluid decreases almost linearly with shorter engine length—from 65 s with a 15 μm Zn compartment to 43 and 8 s with 9 and 3 μm compartments, respectively. UV–vis spectra revealed that increasing the length of the Zn-loaded compartment reduces cargo capacity, as shown in Figure S4 and Figure S8d. Microrockets with an 8 μm Zn compartment were employed for subsequent in vitro and in vivo experiments, achieved by balancing their lifetime (38 s) and cargo loading capacity (50 mg/g).

## Sustained Drug Release from MOFs

Given the diminutive size of therapeutic molecules like amoxicillin, measuring just a few angstroms across, ZIF-8 with porous cavities that have a diameter of about 12 Å and small pore opening of 3.4 Å was chosen as the drug carrier.^49^ Rhodamine 6G (R6G) was utilized as a model drug to trace the drug distribution and drug delivery efficiency. SEM and TEM images of fabricated ZIF-8 and R6G@ZIF-8 particles confirm the uniformity and polyhedral shape of these particles, maintaining a consistent size (Figures 3c and 3d and Figure S5). ImageJ analysis of SEM images revealed that loading R6G had minimal impact on the size or morphology of ZIF-8, with particle sizes averaging 90 to 95 nm (Figure 3e). Dynamic light scattering further validated these findings (Figure S6). These measurements unveiled that the hydrodynamic size distributions of pure ZIF-8 particles (5 mg/mL suspended in distilled water) are similar to those of R6G@ZIF-8 (5 mg/mL suspended in distilled water). X-ray diffraction confirmed the high crystallinity of ZIF-8 and R6G@ZIF-8, matching standard ZIF-8 crystals (Figure 3f), and nitrogen adsorption–desorption isotherms displayed a typical type-I curve, indicating high microporosity with a BET surface area of 1313.21 m^2^/g (Figure 3g). This combination of small pore size and large surface area underscores ZIF-8’s suitability as a drug carrier. The highest encapsulation rate of R6G@ZIF-8 achieved was calculated as 92%, obtained with the optimized concentration of R6G (1 mg/mL) as shown in Figure S8c.

The sustained drug release capability of the MOF-based microrockets was investigated by measuring the release rate of R6G from both ZIF-8@gelatin and bare gelatin into phosphate buffered saline (PBS) with a pH of 6.8 and heated at 37 °C, simulating the condition in the mucosal layer. The release percentages of R6G were calculated according to the following formula: release percentage (wt %) = *m*_i_/*m*_0_, where *m*_i_ is the amount of released R6G and *m*_0_ is the total amount of loaded R6G. The release pattern showed an initial rapid release from bare gelatin, ceasing within 2 days, whereas ZIF-8 facilitated two peak releases before stabilizing, extending the release duration up to 15 times longer than bare gelatin (Figure 3h). The first increase of release rate resulted from desorption of R6G from ZIF-8 and the second increase marks the decomposition of ZIF-8 drug carriers. This indicates the potential of ZIF-8 microrockets for sustained drug delivery.

## In Vitro and Toxicity Study

**Figure 4:**
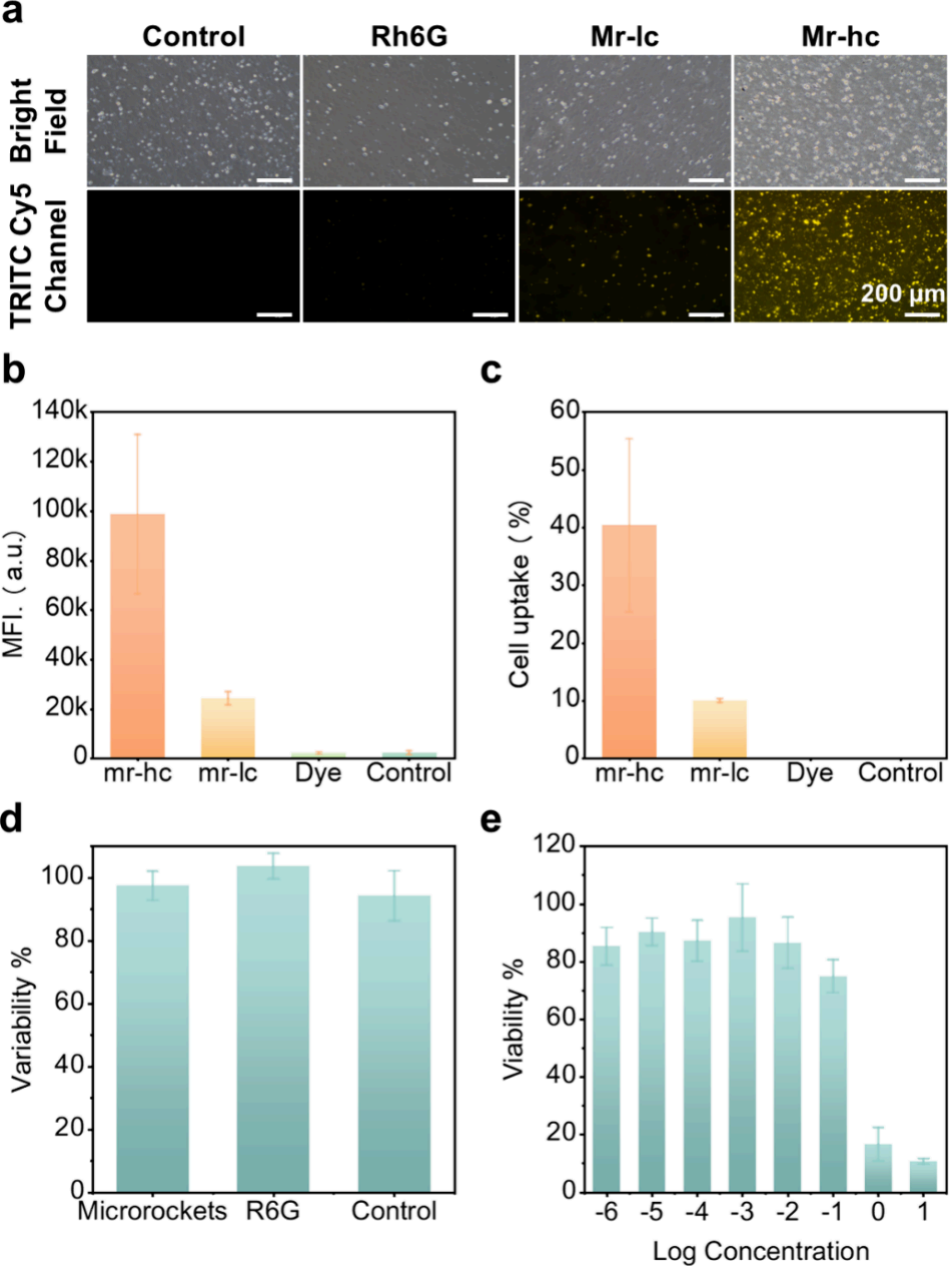
In vitro testing of microrockets. (a) Microscopic images of NCI-N87 gastric epithelial cells treated with pure mucin, mucus with Rh6G, and mucus with microrockets at a low concentration (mr-lc, 0.2 mg/mL) and a high concentration (mr-hc, 2 mg/mL), respectively. (b) Mean fluorescence intensity (MFI) of NCI-N87 cells. (c) Drug uptake percentage by NCI-N87 cells. (d) Viability of NCI-N87 cells treated with microrockets, R6G, and a no-treatment control, respectively. (e) Viability of NCI-N87 cells treated with microrockets across a range of concentrations.

The controlled drug release capability of the microrockets was assessed using a monolayer of NCI-N87 epithelial cells, cultured in membrane inserts to form a monolayer, followed by the addition of mucin and stimulated gastric acid to mimic the environment of the stomach. The monolayer integrity of NCI-N87 epithelial cells was then treated with pure PBS, R6G solution, a suspension of low-concentration MOF-based microrockets (mr-lc, 0.2 mg/mL), and a suspension of the high-concentration microrockets (mr-hc, 2 mg/mL), respectively, for 24 h (Figure S10). Bright-field and fluorescent microscopy showed no background fluorescence with pure mucin and no drug penetration with R6G solution, whereas even low concentrations of microrockets (0.2 mg/mL) achieved drug delivery to the epithelial cells (Figure 4a).

The uptake efficacy of R6G by the NCI-N87 epithelial cells was quantified by measuring the mean fluorescent intensity (MFI) of the cells and the number of cells exhibiting a fluorescent signal using flow cytometry (Figures 4b and 4c). These results confirm that only Zn-powered microrockets could successfully deliver the model drug to the cell monolayer and a higher concentration of microrockets contributed to a higher R6G uptake efficiency. Up to 40% of the epithelial cells ingested the model drug at a microrocket concentration of 2 mg/mL and about 10% of cells ingested the drug at a concentration of 0.2 mg/mL. These findings demonstrate that the microrockets possess the ability to transport drugs into the mucosal layer of the stomach, enabling controlled drug release.

An acute toxicity study confirmed the biosafety of the microrockets, showing no significant cytotoxic effects at various concentrations compared to controls (Figures 4d and 4e). The cell viability showed no statistically significant difference for the experiments with microrockets of 2 μg/mL, pure R6G with the same amount, and the no-treatment control (Figure 4d), indicating that the microrockets had no obvious cytotoxicity to the NCI-N87 gastric epithelial cell line. Moreover, the microrockets exhibited good biocompatibility at a wide range of concentrations from 2 × 10^–6^ to 2 × 10^–1^ mg/mL (Figure 4e). The microrockets’ biocompatibility, attributed to nontoxic degradation products like Zn^2+^ and 2-MeIm^50^ and the use of biocompatible materials such as PEDOT and Au, supports their safe use in drug delivery.^51^

## In Vivo Distribution Study

A mouse model was employed to conduct an in vivo retention study, assessing the controlled and sustained release capabilities of the microrockets in an authentic gastric environment. All of the animal procedures were approved by the laboratory animal facility (CWB) in Hong Kong University of Science and Technology (AEP-2022-0056) and were performed under general anesthesia. IRDye 800CW carboxylate (IR800CW) was utilized as a model drug for scanning the drug distribution in different tissues. Prior to the experiment, all mice were starved overnight to eliminate any potential food interference. Then they were separated into three groups that were fed with IR800CW@ZIF-based microrockets (PEDOT/Au/Zn/IR800CW@ZIF-8) and the free model drug of IR800CW.

**Figure 5:**
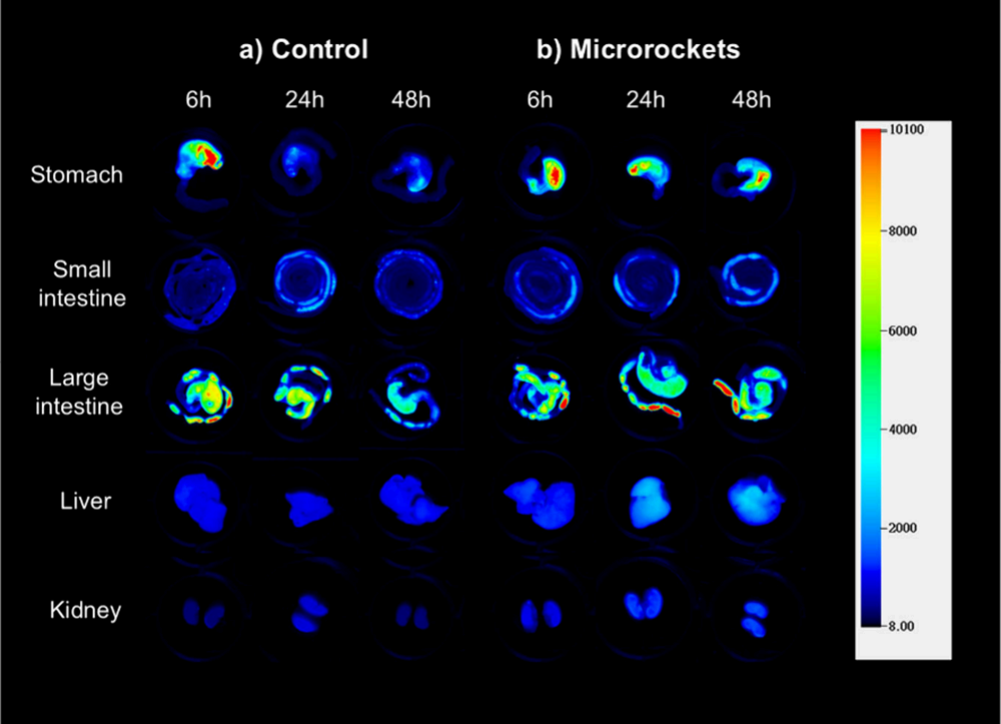
Body retention of the single model drug and drug-laden microrockets: in vivo NIRF images of five organs (stomach, small and large intestine, liver, and kidney) after oral administration with (a) single IRdye 800CW dye as model drug after 6, 24, and 48 h (Control) and (b) IRdye 800CW dye encapsulated in MOF-based microrockets after 6, 24, and 48 h (Microrockets).

The infrared signal of the stomach, small intestine, large intestines, liver, and kidneys of these mice were measured at 6, 24, and 48 h of postoral administration (Figure 5 and Figure S12). The results clearly indicated that the stomachs exhibited significantly high signals following the administration of microrockets overtime, which suggests prolonged stomach retention. Notably, the signals remain strong even after 48 h of administration, confirming the controlled and sustained drug delivery to the gastric mucus layer facilitated by the MOF-based microrockets. In contrast, the control group displayed a rapid decline in signal intensity over time after 24 h of administration, indicating that the concentration of the free model drug fell below the effective threshold. And at 72 h, both the model drug concentrations with and without the microrockets demonstrated an obvious decrease, indicating the conclusion of the model drug release process in the stomach (Figure S12). This comparison underscores the enhanced performance and superior advantages of our microrocket-based drug delivery system.

An innovative drug delivery system using Zn-powered, MOF-based microrockets was developed to target the gastric mucosal layer, enabling controlled and sustained medication release. These microrockets have a cylindrical shape of 15 μm in length and 5 μm in diameter and are propelled by redox-reaction-generated bubbles with a speed up to 40 μm/s in acid. The lifespan of these microrockets can be adjusted from 10 to 70 s by modifying the length of the Zn-loaded compartment. The high porosity of MOFs significantly extends drug release up to 1 month in the simulated gastric mucosa, which is 10-fold longer than that achieved with pure drugs. The sustainability and control over the drug release are further ensured by the pH-sensitive enteric coating, which shields the MOFs from harsh acids while triggering drug release at the neutral pH of the mucosal layer. A drug retention exceeding 48 h in the stomach of mice was demonstrated in our in vivo experiments. Overall, our proposed drug delivery system provides a promising solution for controlled and sustained drug release in challenging environments. It holds the potential for diverse biomedical applications, notably in treating conditions such as *Helicobacter pylori* infections and diabetes and facilitating the oral delivery of biomacromolecules.

## Supporting Information Available

Figures S1–S12 showing additional experimental results (PDF)
Video S1 showing propulsion of self-propelled microrockets in simulated gastric fluid (MP4)
