# Supplementary material for: Metal–Organic Frameworks-Based Microrockets for Controlled and Sustained Drug Release
Source: Nano Lett. 2025 Mar 17;25(15):5989–96. doi: 10.1021/acs.nanolett.4c04628 (PMC12007105; doi:10.1021/acs.nanolett.4c04628)
Supplement: Supplementary file 1 — nl4c04628_si_001.pdf [file nl4c04628_si_001.pdf]

Metal-Organic Framework-Based Microrockets for Controlled and Sustained Drug Release

Supplementary information

Zixi Wan‡[1,3], Casper H.Y. Chung‡[1], Chi Ming Laurence Lau[2], Jin Teng Chung[2], Ying Chau[2], Zhiyong Fan[2,3], Shuaizhong Zhang[1], Shuhuai Yao[1,2]*

1 Department of Mechanical and Aerospace Engineering, The Hong Kong University of Science and Technology, Hong Kong SAR, China

2 Department of Chemical and Biological Engineering, The Hong Kong University of Science and Technology, Hong Kong SAR, China

3 Department of Electronic and Computer Engineering, The Hong Kong University of Science and Technology, Hong Kong SAR, China

Email: meshyao@ust.hk

Contents

1. Materials and Methods
2. Supporting Video Description

   Supporting video S1. Propulsion of self-propelled microrocket in simulated gastric fluid

3. Supporting Figures

   Figure S1. SEM image and EDX line scan of Zinc (Zn) and Carbon (C).

   Figure S2. a) The morphology of microrockets at different fabrication steps: 1) PEDOT/Au, 2) PEDOT/Au/Zn, 3) PEDOT/Au/Zn/R6G@ZIF-8/Enteric coating; b) SEM image with EDX of the fabricated microrockets (top view).

   Figure S3. Photos of the well-suspended micromotors in an EtOH solution.

   Figure S4. UV-Vis spectra of the microrockets with Zn of different lengths: 1) No zinc, 2) 8μm, 3) 15μm.

   Figure S5. SEM image of pure ZIF-8 particles.

   Figure S6. Hydraulic diameter distribution by dynamic light scattering (DLS) data analysis of ZIF-8, R6G@ZIF-8_1 (1:1 w/w), and R6G@ZIF-8_2 (4:1 w/w).

Figure S7. Model drug (R6G) release rate under various pH conditions

Figure S8. a) UV-Vis spectrum and b) standard curves of standard R6G solutions from 1 μg/mL to 20 μg/mL in DI water; c) encapsulation rate (EN%) of ZIF-8 based different R6G concentration on; d) drug loading capacity of microrockets based on the capacity length.

Figure S9. Confocal microscopy images of a-c) R6G@ZIF-8 suspension; d-f) fabricated MOF-based microrockets without any treatment; g-i) microrockets treated with stimulated gastric acid (pH=1.2); j-l) microrockets treated with neutral PBS solution (pH=6.8). Among the confocal images, the left column shows the merged images, the middle column shows the view of bright field, and the right column shows the fluorescent images.

Figure S10. Schematic illustration of the simulated gastric environment for in vitro and toxicity studies.

Figure S11. Photo of the organs for in vivo distribution study.

Figure S12. Stomach retention of the single model drug and drug-laden microrockets: In vivo NIRF images of mice's stomachs after oral-administrated with a) single IRdye® 800CW dye as model drug after 6, 24, 48, and 72 hours (Control); b) IRdye® 800CW dye encapsulated in MOF-based microrockets after 6, 24, 48, and 72 hours (Microrockets).

Materials and methods

Fabrication of microrockets. The multicompartmental microrockets were fabricated using a template-based electrodeposition method.[50,51,58] The used template was transparent polycarbonate membrane (PCM) featured with pores of 5 μm in diameter (Catalog No. 7060-2513; Whatman, Maidstone, UK). Briefly, there were six fabrication steps. 1) An Au layer of 75 nm was sputtered on one side of the PCM at room temperature under a vacuum of 4×10^{-6} torr with a DC power of 200W using a Denton Discovery 18 sputtering system (Moorestown, NJ, USA). This Au layer served as the working electrode for the electrodeposition of the PEDOT/Au micro-shell. 2) The PEDOT/Au shell was electrodeposited within the micropores by assembling the PCM template in a three-electrode cell in an electrochemical workstation (CHI 660D; Champaign, IL, USA), with an aluminum foil contacting the Au film, a Pt wire and Ag/AgCl with KCl serving as the working, counter and reference electrode, respectively. The PEDOT shell was electrodeposited at a constant voltage of +0.8 V for a certain time until the total electric quantity reached 0.12 C, such that the final PEDOT walls were approximately 200 nm thick. The plating solution for PEDOT consisted of 15 mM 3,4-ethylenedioxythiophene (EDOT) monomer, 50 mM sodium dodecyl sulfate (SDS) surfactant and 7.5 mM potassium nitrate (KNO_3) electrolyte. The inner Au tube was electrodeposited at -0.9 V for 0.9 C with a commercial gold plating solution. 3) The Zn segment was then electrodeposited galvanostatically at a constant electric current of -6 mA from a Zn plating solution containing 68 g/L zinc chloride ($ZnCl_2$) and 20 g/L boric acid (H_3BO_3) (buffered to pH = 2.5 with sulfuric acid). The

deposition efficiency of Zn was approximately 0.8 μm/min. After Zn deposition, excess Au film and overflowed Zn were removed by ion beam milling for 20 min under 200 V of screen voltage with a milling angle of 60°. This step was undertaken to increase the purity of the microrockets suspension and avoid interference from impurities such as gold and excess zinc after PCM dissolution. The completion of removal process was indicated by visually inspecting the membrane, which changed in color from yellow to transparent. 4) A mixture of drug-encapsulated ZIFs and gelatin was transfused into the template which was stored at 4 °C overnight. 5) A pH-sensitive enteric coating was made of Eudragit L100 polymer (8 mg/mL in ethanol, EVONIK Company) spreading over the PCM with excessive solution and removed immediately.[52] The sample was placed at room temperature for 2 hours to harden the protective enteric polymer film. 6) The PCM template was dissolved using dichloromethane (DCM, anhydrous, purity ≥ 99.8%, Sigma-Aldrich) for 20 mins to fully release the microrockets. The microrockets were then collected by centrifugation at 6500 rpm for 3 mins and washed with DCM, isopropanol, and ethanol three times each. All microrockets were stored in deoxygenated ethanol at 4 °C for further use (Figure S3). All used chemicals were purchased from Sigma-Aldrich.

Synthesis of drug-loaded ZIF-8. We used IRDye® 800CW carboxylate as a model drug for scanning the drug distribution in tissue for the *in vivo* experiments and used Rhodamine 6G (R6G) as a model drug to trace the drug distribution and drug delivery efficiency. Here we encapsulated the model drug in ZIFs by a one-pot synthesis, which is simple and yields a high encapsulation rate of the target drug molecules (Figure S8).[54]

Firstly, R6G solutions (99%, Sigma-Aldrich) of 5 mg·mL^{-1} were prepared in methanol and deionized water, respectively, for further use. 0.04 g of $Zn(NO_3)\cdot 6H_2O$ was dissolved in 8 ml of methanol. 4 ml of R6G (1 mg·mL^{-1}) in methanol was then added into the $Zn(NO_3)_2$ solution. The mixture of Zn^{2+} and R6G was stirred for 5 mins, followed by the addition of 80 ml of a methanol solution containing 0.28 g of 2-methylimidazole (2-MeIm) (99%, Sigma-Aldrich), which was stirred for 15 mins. The precipitated R6G@ZIF-8 nanoparticles were collected by centrifugal separation at 10000 rpm for 10 min, rinsed three times, and then dried in the oven at 60°C for overnight. Subsequently, R6G@ZIF-8 particles (5 mg/mL in distilled water) were added into gelatin solution (25 mg/mL in distilled water) and heated to 50 °C under stirring.[54]

Sustained drug release from ZIF-8 nanoparticles. 50 mg of R6G@ZIF-8 nanoparticles were suspended in 10 mL gelatin which was heated up to 50 °C to make it from gel to liquid state. 30 μL of the suspension was added into 970 μL of PBS solution (pH=6.8). The mixture was then maintained at 37 °C during the whole release test. 1 mL of the release medium was sampled at each time point. A varioskan lux multimode microplate reader (Thermo Fisher Scientific) was used to determine the quantity of R6G that had been released from ZIF-8. We also measured the release rate of pure R6G of the same concentration in gelatin as a control to illustrate the sustainable release by ZIF-8.

In vitro study of controlled drug delivery and biocompatibility. NCI-N87 gastric epithelial cells (ATCC, P2, 1 x 10^5 cells/well) were seeded on a Transwell® insert

(Costar® Corning Incorporated, 5.0 µm pore, 6.5 mm diameter) and cultured for 7 days prior to treatment to establish a cell monolayer. The medium was changed each day. 3.4 µL of complete culture medium was added at the lower chamber of the Transwell® insert. During treatment, 5 µL of mucin was added on top of the cell monolayer, and 100 µL simulated gastric acid was added on top of mucin layer. Then 80 µL of R6G and the fabricated MOF-based microrockets with a concentration of 2 mg/mL and 0.2 mg/mL in 1X PBS were added into the gastric fluids, respectively. Cells were treated for 24 hours, and subjected to fluorescence microscopy (20X magnification, Nikon Eclipse Ti2-E) and flow cytometry (BD FACS Aria III Cell Sorter and Analyzer). Briefly, mucin and simulated gastric acid were removed. Cells were trypsinized and resuspended in ice-cold PBS, then washed with ice-cold PBS followed by 0.0001% Triton-X, and finally resuspend in PBS for fluorescence microscopy imaging and flow cytometric analysis. For cytotoxicity test of microrockets, NCI-N87 cells (P2, 1 x 10^5 cells/well) were treated with microrockets of different concentrations (from 2 x 10^{-6} to 20 mg/mL) and R6G, respectively. The cell viability was quantified with the flow cytometry (BD FACS Aria III Cell Sorter and Analyzer).

In vivo study of controlled and sustained drug release. Before the animal handling and administration, free IR800CW dye solution and IR800CW dye encapsulated microrocket solution were prepared in deionized distilled water (DDI water). The mass concentration of the dye was normalized to 20 ng/mL for both solutions to ensure consistent dosing across the experimental groups and different batches of microrockets. C57B6 mice, at least 6 months old and of random sex, were used in this study. The

mice were fed with whole grain bread, which yields minimal background IR800CW signal to ensure accurate measurement of the administered dye. Prior to the experiment, the mice were starved overnight while maintaining free access to water. Mice were orally fed with either the free IR800CW dye solution or the IR800CW dye encapsulated MOF-based microrockets suspension using a 0.5 mm PTFE soft gavage needle (LOCTITE®). Each mouse received 0.1 mL of the respective solution, equivalent to 2 ng of IR800CW dye per mouse. Following the oral administration, the mice were given free access to whole grain bread and water. At designated time points (6/24/48 hours post feeding), the mice were sacrificed by cervical dislocation. The oesophagus, stomach, small intestine, large intestine, liver, and kidney were immediately harvested and placed in ordinary polystyrene, flat-bottom 6-well plates (Figure S11). The organs were then scanned using the LI-COR Biosciences Odyssey Infrared Imaging System (LI-COR Biosciences) to measure the signal at an excitation wavelength of 800 nm. The obtained images were analyzed using Image Studio™ Lite Software 5.5 (LI-COR Biosciences) to quantify the distribution and intensity of the IR800CW dye in the harvested tissues.

Stability study of drug release under various pH conditions. The stability of the enteric coating under strong acidic conditions was evaluated by measuring the quatity of the model drug, R6G, released from ZIF-8 in gelatin covered with the enteric coating at various pH levels (pH = 1, 3, 6, and 7). 30 μL of gelatin containing R6G@ZIF-8 (5 mg/mL) was stored at 4 °C overnight before being coated with a solution of 25 mg/mL Eudragit L100 polymer in ethanol. The enteric coating was then hardened at room

temperature for 2 hours. Following this, the samples were treated with 970 μL of simulated gastric fluid (pH=1 and 3) and PBS solutions (pH= 6 and 7), respectively. The mixtures were maintained at 37 °C throughout the release test. Samples of the release medium were collected at designated time points to measure the quantity of R6G released, which was determined using a Varioskan Lux multimode microplate reader (Thermo Fisher Scientific). To eliminate the impact of pH on fluorescence intensity, all supernatant solutions were adjusted to neutral pH using sodium hydroxide (NaOH). As illustrated in Figure S7, the model drug exhibits minimal release into the surrounding media under acidic conditions (pH = 1 or 3) when coated with the enteric polymer. However, when the pH is increased to 6 and 7, the drug is successfully released and diffuses into the neutral media. This observation demonstrates the stability of the enteric coating in strong acidic environments.

Encapsulation rate and drug loading rate measurement. Initially, solutions of R6G at various concentrations (1-20 μg/mL) were prepared and analyzed using UV-Vis spectroscopy to establish the standard curves (Figure S8a and b). To quantify the drug loading in ZIF-8, the supernatant solutions of the R6G@ZIF-8 after rinsing were collected. The R6G@ZIF-8 samples were fabricated as described in the Methods section, utilizing different concentrations of R6G as the model drug. Using the standard curves of R6G solutions for quantifying the supernatant, the highest encapsulation rate of R6G@ZIF-8 achieved was 92%, obtained with the optimized concentration of R6G (1mg/mL) (Figure S8c). Ignoring the loss of gel during infiltration into the microrockets, the results can also be regarded as the encapsulation rate of the entire system. The drug

loading capacity of the microrockets is determined by the length of Zn-loaded compartment. Specifically, longer length of Zn-loaded compartment result in a reduced drug loading capacity (Figure S4). We quantified the amount of the model drug in the microrocket suspension using a plate reader (Figure S8d). The drug loading capacity of the microrockets with an 8 μm length of Zn-loaded compartment is approximately 50 mg/g.

Below are the methods for measuring the encapsulation rate and drug loading capacity. The encapsulation rate (EN%) was calculated using:

$$EN\%=(W_t/W_i)\times 100\%$$

where W_t represents the quantity of the molecules in ZIF-8, and W_i represents the initial quantity of molecules added.

And the drug loading capacity of the microrockets is calculated using the formula:

$$\text{Drug loading capacity} = m_d/m_0$$

where m_d represents the weight of drug loaded, and m_0 represents the total weight of the drug-loaded microrockets.

Material characterization. Morphology of the microrockets and MOFs was characterized by scanning electron microscope (SEM, JSM-7100F, JEOL) and aberration-correction transmission electron microscope (STEM, JEM-ARM200F, JEOL). EDX mapping was achieved by scanning electron microscope (SEM, JSM-7100F, JEOL). Motion of microrockets were filmed by the camera (Nikon D200) with microlens. Bright-field, fluorescence, and merged images of the fabricated microrockets were obtained with a confocal microscope (Zeiss LSM 980 Confocal

Microscope with AiryScan 2). Element characterization was done by X-ray diffraction (XRD, X'pert Pro (PANalytical)) with scanning angle from 5-30.

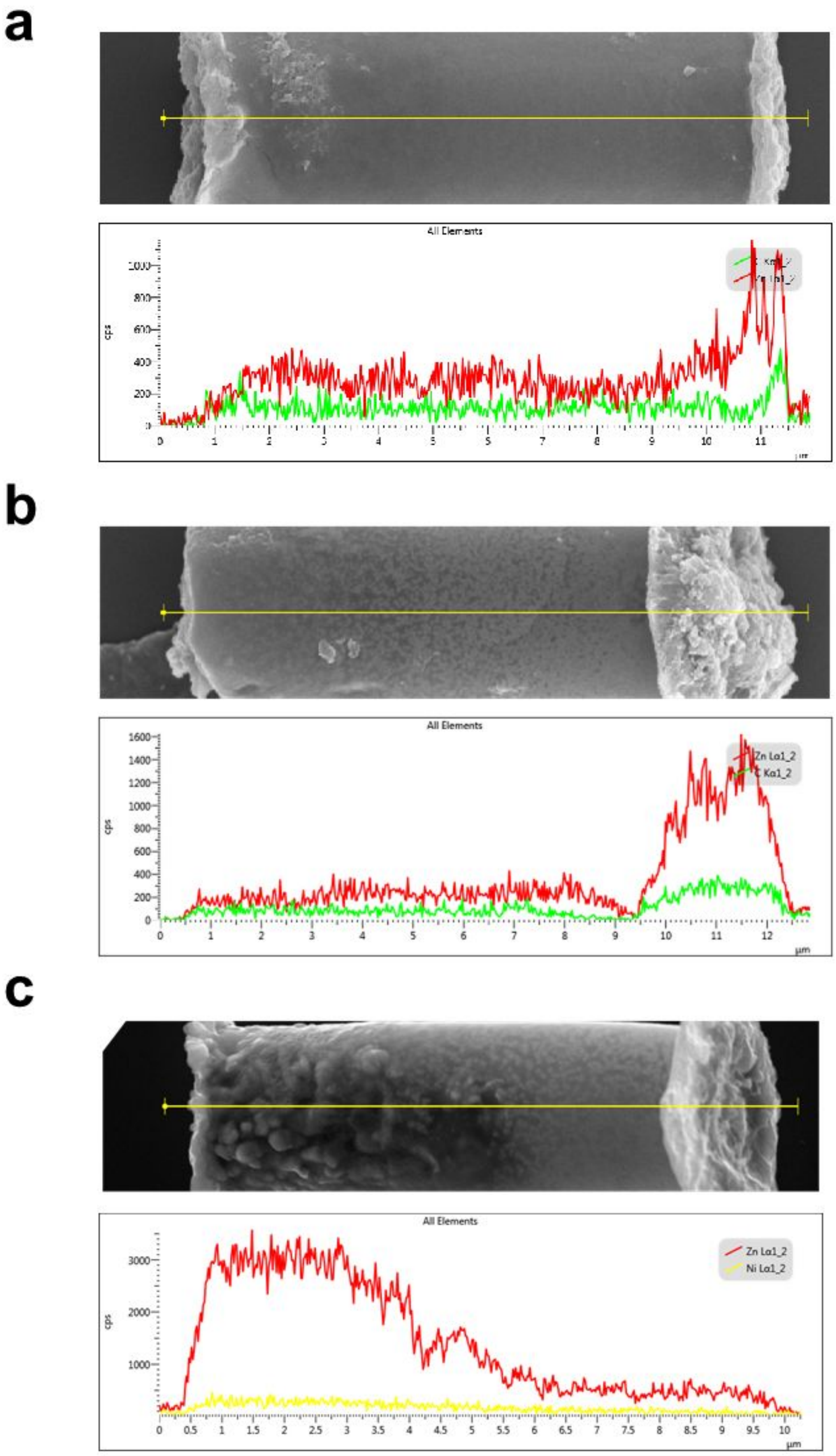

Figure S1. SEM image and EDX line scan of Zinc (Zn) and Carbon (C).

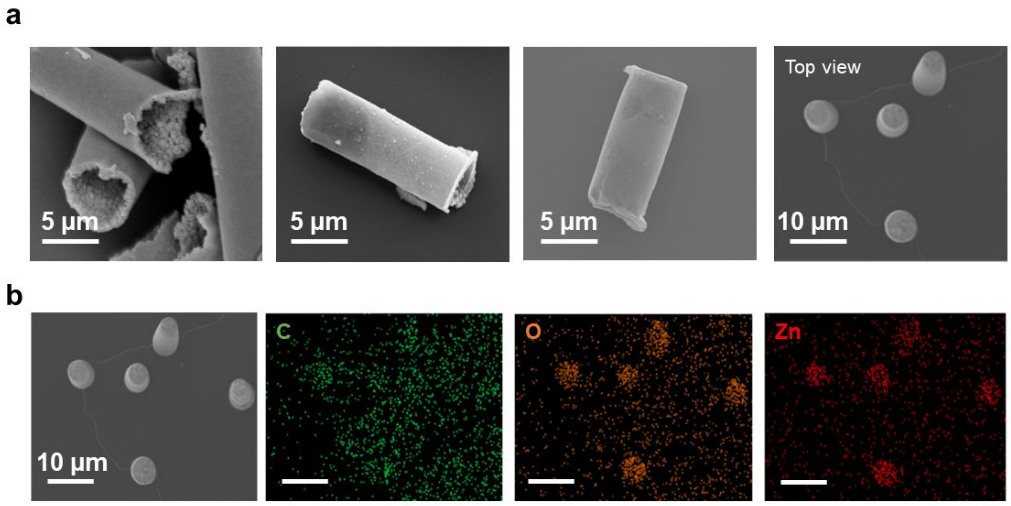

Figure S2. a) The morphology of microrockets at different fabrication steps: 1) PEDOT/Au, 2) PEDOT/Au/Zn, 3) PEDOT/Au/Zn/R6G@ZIF-8/Enteric coating; b) SEM with EDX of the fabricated microrockets (top view).

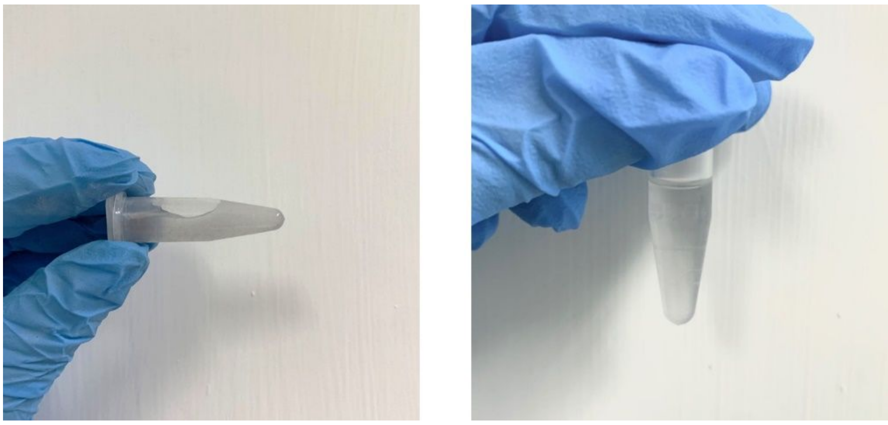

Figure S3. Photos of the well-suspended micromotors in an EtOH solution.

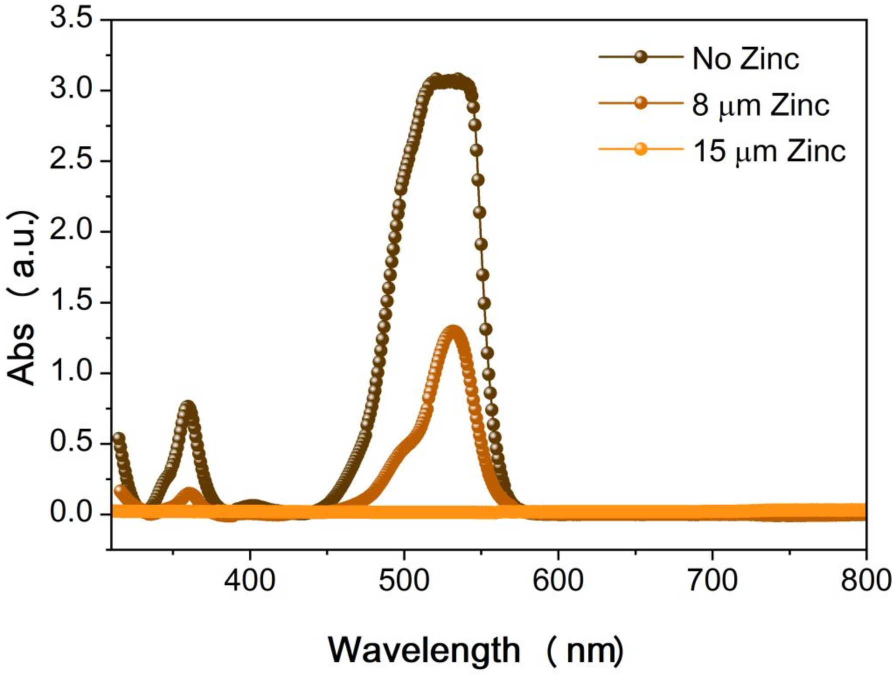

Figure S4. UV-Vis spectra of the microrockets with Zn of different lengths: 1) No zinc, 2) 8μm, 3) 15μm.

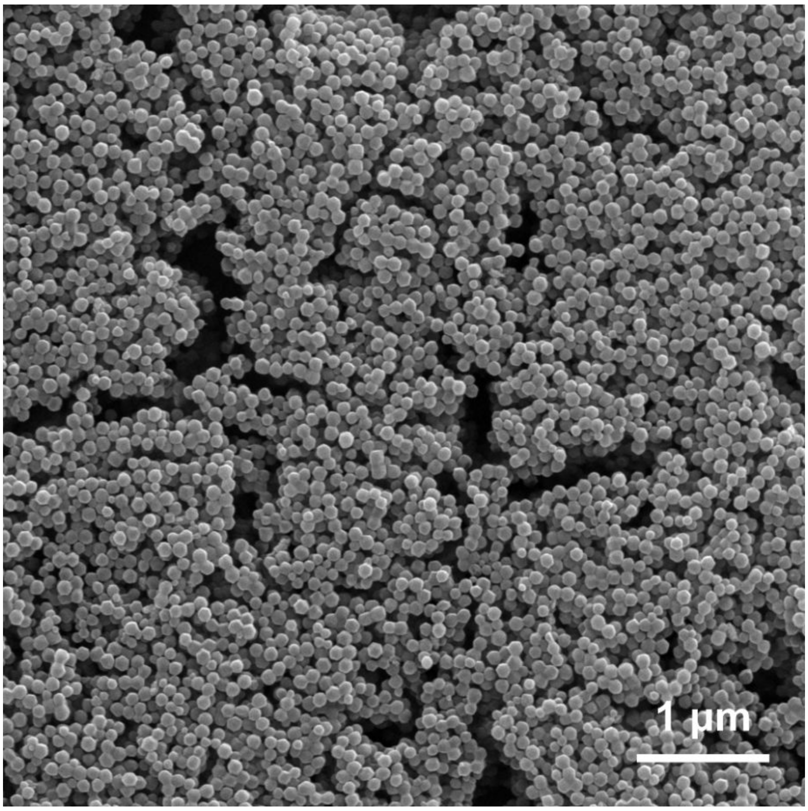

Figure S5. SEM image of pure ZIF-8 particles.

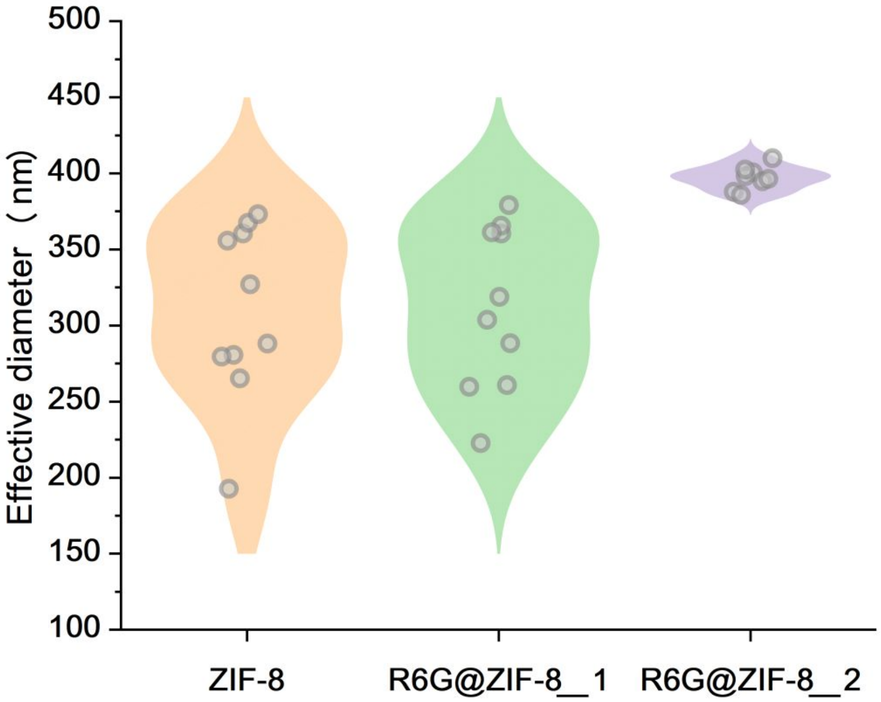

Figure S6. Hydraulic diameter distribution by dynamic light scattering (DLS) data analysis of ZIF-8, R6G@ZIF-8_1 (1:1 w/w), and R6G@ZIF-8_2 (4:1 w/w).

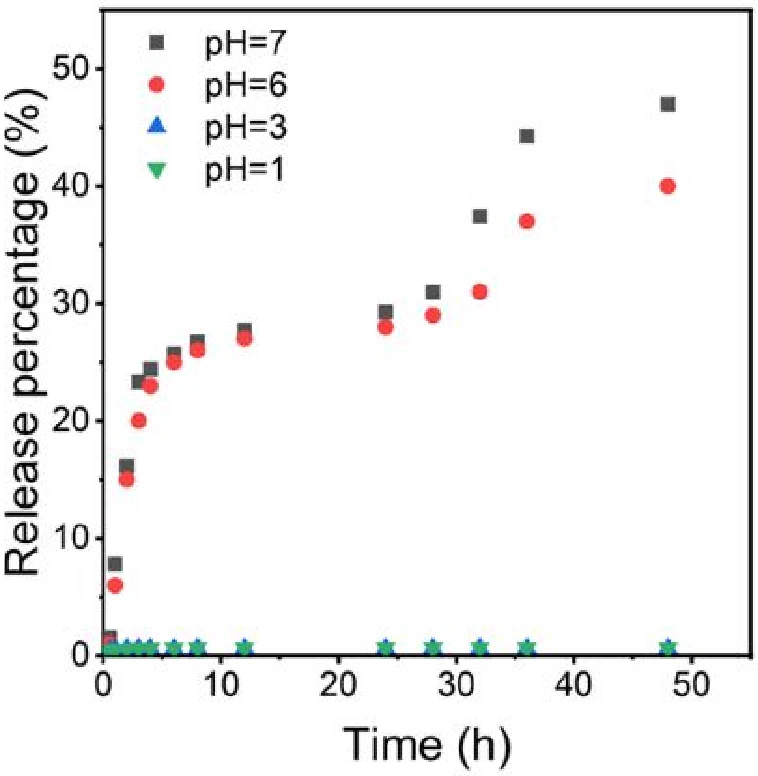

Figure S7. Model drug (R6G) release rate under various pH conditions

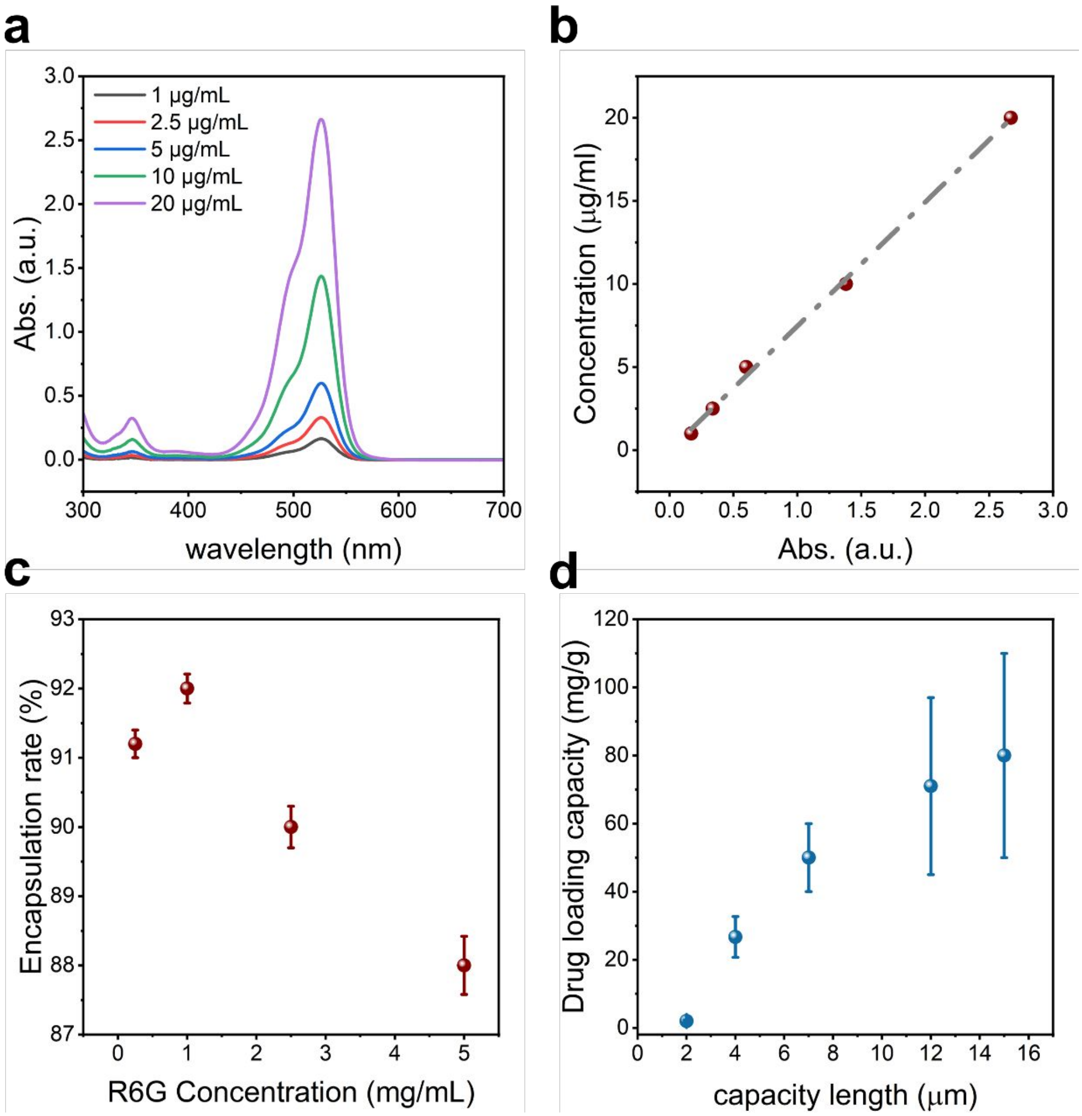

Figure S8. a) UV-Vis spectrum and b) standard curves of standard R6G solutions from 1 μg/mL to 20 μg/mL in DI water; c) encapsulation rate (EN%) of ZIF-8 based different R6G concentration on; d) drug loading capacity of microrockets based on the capacity length.

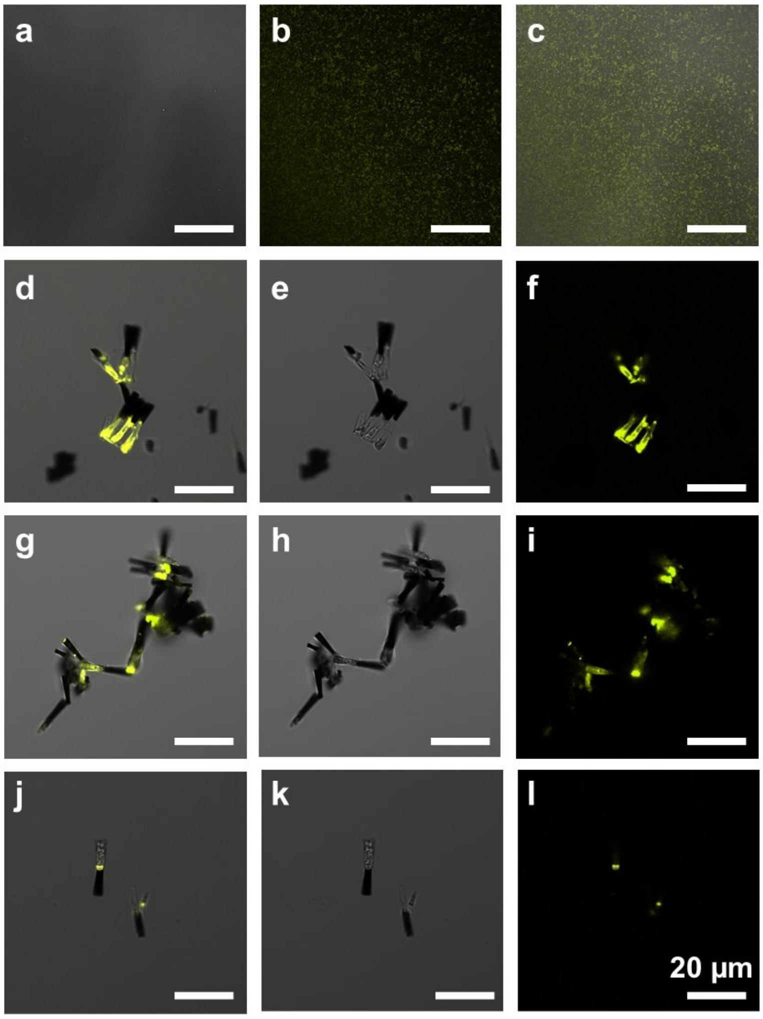

Figure S9. Confocal microscopy images of a-c) R6G@ZIF-8 suspension; d-f) fabricated MOF-based microrockets without any treatment; g-i) microrockets treated with stimulated gastric acid (pH=1.2); j-l) microrockets treated with neutral PBS solution (pH=6.8). Among the confocal images, the left column shows the merged images, the middle column shows the view of bright field, and the right column shows the fluorescent images. (scale bar: 20μm)

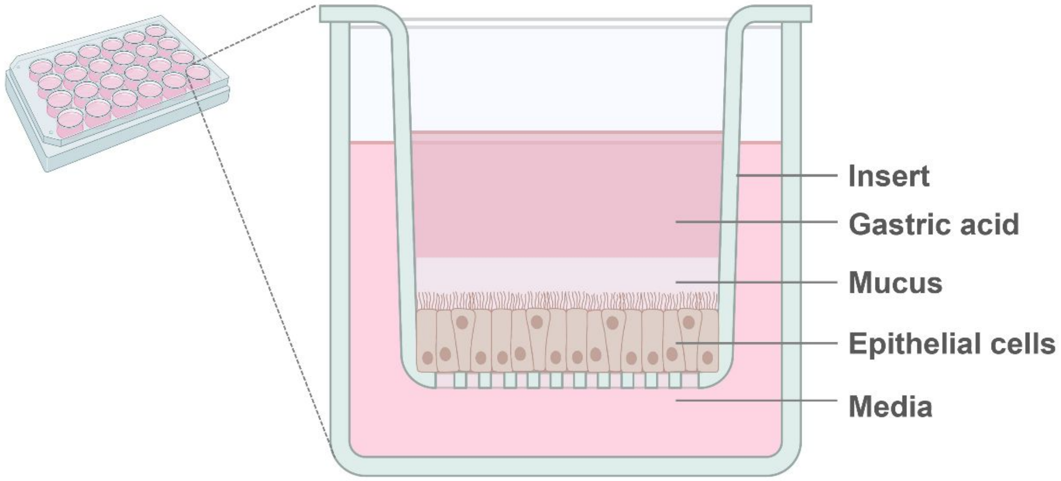

Figure S10. Schematic illustration of the simulated gastric environment for in vitro and toxicity studies.

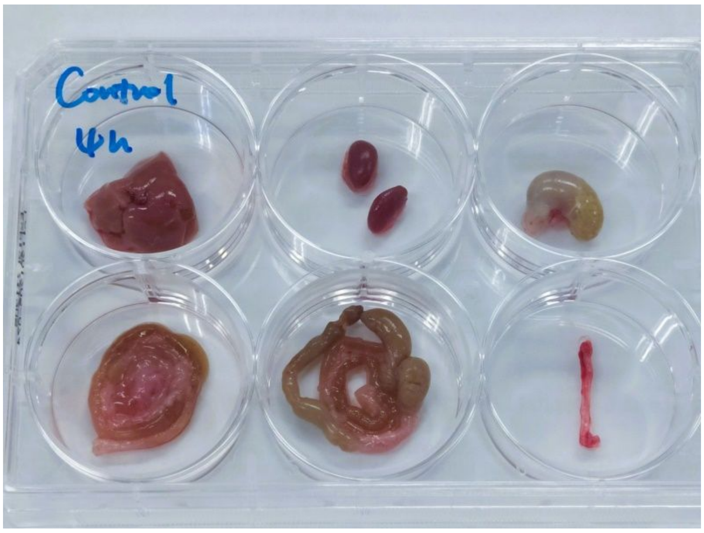

Figure S11. Photo of the organs for in vivo distribution study.

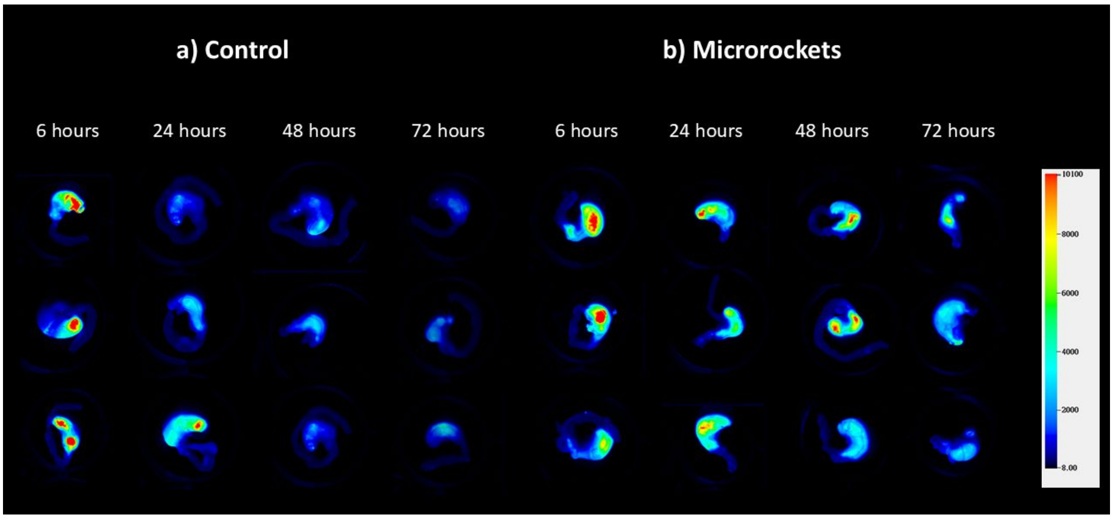

Figure S12. Stomach retention of the single model drug and drug-laden microrockets: In vivo NIRF images of mice's stomachs after oral-administrated with a) single IRdye® 800CW dye as model drug after 6, 24, 48, and 72 hours (Control); b) IRdye® 800CW dye encapsulated in MOF-based microrockets after 6, 24, 48, and 72 hours (Microrockets).
